# Toll-like receptor 4 signaling activates ERG function in prostate cancer and provides a therapeutic target

**DOI:** 10.1093/narcan/zcaa046

**Published:** 2021-01-27

**Authors:** Benjamin M Greulich, Joshua P Plotnik, Travis J Jerde, Peter C Hollenhorst

**Affiliations:** Medical Sciences, Indiana University School of Medicine, Bloomington, IN 47405, USA; Biology Department, Indiana University, Bloomington, IN 47405, USA; Department of Pharmacology and Toxicology, Indiana University School of Medicine, Indianapolis, IN 46202, USA; Medical Sciences, Indiana University School of Medicine, Bloomington, IN 47405, USA

## Abstract

The *TMPRSS2–ERG* gene fusion and subsequent overexpression of the ERG transcription factor occurs in ∼50% of prostate tumors, making it the most common abnormality of the prostate cancer genome. While ERG has been shown to drive tumor progression and cancer-related phenotypes, as a transcription factor it is difficult to target therapeutically. Using a genetic screen, we identified the toll-like receptor 4 (TLR4) signaling pathway as important for ERG function in prostate cells. Our data confirm previous reports that ERG can transcriptionally activate TLR4 gene expression; however, using a constitutively active ERG mutant, we demonstrate that the critical function of TLR4 signaling is upstream, promoting ERG phosphorylation at serine 96 and ERG transcriptional activation. The TLR4 inhibitor, TAK-242, attenuated ERG-mediated migration, clonogenic survival, target gene activation and tumor growth. Together these data indicate a mechanistic basis for inhibition of TLR4 signaling as a treatment for ERG-positive prostate cancer.

## Graphical Abstract

**Graphical Abstract:**
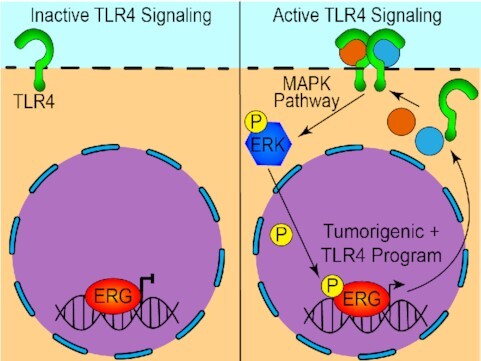
Toll-like receptor 4 signaling activates ERG function in prostate cancer and provides a therapeutic target.

## INTRODUCTION

Prostate cancer is characterized by a high prevalence of fusions between genes with active promoters and genes encoding ETS family transcription factors. The most common example is the gene fusion between the promoter and 5′ UTR of the androgen-driven *TMPRSS2* gene and the ETS transcription factor, *ERG* (1). This results in androgen-dependent overexpression of ERG protein, which drives cellular migration (2,3). Furthermore, ERG expression, coupled with either PI3K/AKT activation, usually through *PTEN* deletion, or loss of FOXO1, drives the formation of prostatic adenocarcinoma (4,5). While anti-androgen treatments are initially efficacious, patients often develop resistance to these treatments and become castration-resistant (6,7). This stage is often accompanied by the development of metastatic lesions and greatly increased mortality (8,9). Few treatments exist for patients with this advanced stage of disease, which has created a need for new therapeutics, particularly those independent of androgen receptor.

Toll-like receptor 4 (TLR4) is a transmembrane receptor that is traditionally expressed on immune cells such as macrophages, where it recognizes lipopolysaccharide, a component of Gram-negative bacterial outer membranes. This allows TLR4 to act as a sensor for bacterial infection and transduce a signal to activate the inflammatory response through NF-κB (10). However, there have been numerous reports in the literature suggesting a TLR4 role in carcinomas, including pancreatic, colorectal, lung, breast, ovarian and prostate cancer (11–14). In ovarian cancer, LPS-induced activation of TLR4 can drive cellular proliferation, and TLR4 knockdowns demonstrate a loss of paclitaxel resistance (15). Stimulation of TLR4 can also increase expression of immunosuppressive cytokines and provide resistance to apoptosis in lung cancer cells (16). In pancreatic cancer, TLR4 activity can promote EMT through M2-polarized tumor-associated macrophages (12). Additionally, non-canonical endogenous ligands of TLR4 can activate TLR signaling independent of bacterial products. One endogenous ligand, BGN, can activate TLR4 in gastric cancer (17). Members of the heat shock protein family, including Hsp70 and Hsc70, have also been implicated as endogenous ligands of TLR4 (18–20).

In prostate cancer specifically, TLR4 knockdown can reduce survival and invasion, and this has been attributed to TLR4’s canonical downstream effectors such as NF-kB (11,21). In 2011, it was demonstrated that ERG can drive expression of TLR4, which then regulates NF-kB activity (22). This has created an ERG–TLR4-NF–kB axis that could contribute to prostate cancer development. While TLR4 has been postulated as a potential therapeutic target for prostate cancer, it has not previously been targeted pharmacologically.

Here we show that a specific TLR4 inhibitor, TAK-242, can disrupt the ERG–TLR4 axis and reduce ERG-mediated phenotypes. This suppression of ERG function extends across both androgen-dependent and androgen-independent cell line models but is specific to ERG-positive cells. Furthermore, we provide evidence to redefine the ERG–TLR4 axis. We confirm that ERG can promote expression of TLR4, and discover that ERG can also upregulate endogenous TLR4 ligands. However, by combining TLR4 inhibition and a phosphomimetic mutant of ERG, our findings indicate that this downstream activation of TLR4 is dispensable for ERG-mediated tumorigenesis. Instead, we show that the critical function of TLR4 is upstream of ERG, where TLR4 signaling promotes phosphorylation of ERG leading to transcriptional activation.

## MATERIALS AND METHODS

### Cell culture and viral transduction

All cell lines were purchased from ATCC and have been authenticated using the PowerPlex 16HS assay. RWPE cells were maintained in Keratinocyte Serum Free Medium (Gibco). PC3 cells were maintained in F12K media. All cells were incubated at 37°C and 5% CO_2_, and all growth media supplemented with 1× penicillin/streptomycin (Corning). Lentiviruses for shRNAs were produced by co-transfecting HEK293T cells with pMDLg/pRRE (Addgene plasmid 12251), pRSV-Rev (Addgene plasmid 12253) and pMD2.G (Addgene plasmid 12259) packaging plasmids as well as the pLKO.1 cloning vector (Addgene plasmid 8453) containing the associated shRNA sequence (Supplementary Table S1). Retroviral overexpression of ERG (NCBI isoform #1), S96E ERG and ERG/myristoylated AKT were created using the method described in (23).

### Migration assays

The *trans*-well migration assay was previously described in (24). In short, Boyden chambers (8 μm pore size; BD Biosciences) were placed in the wells of a 24-well plate filled with 750 µl of serum-containing media. 5.0 × 10^4^ cells suspended in 500 µl of serum-free media then plated into a Boyden chamber. Any necessary drug treatment was performed, and then the cells incubated for 72 h for RWPE cells, or 48 h for PC3 cells. The medium was then aspirated from the Boyden chambers, the internal portion of the membrane was washed with phosphate-buffered saline (PBS) and cotton swabs, and the membrane was stained with Hema 3 staining kit. The membranes dried for 24 h before being plated on microscope slides. Each condition was performed in duplicate, five images were taken per membrane and cells were counted. For scratch assays, cells were grown to confluence in six-well plates, and then the monolayer was scratched with a P1000 pipette tip. The cells were washed with PBS, and fresh medium was added. Images of the scratches were taken at zero and 24 h and the size of each scratch quantified using ImageJ.

### Clonogenic survival assay

A total of 1000 cells were plated in 3 ml of media per well of a six-well plate. The cells incubated for 24 h before drug treatment. The plates were then incubated for another 9 days before the cells were fixed with 10% formalin and stained with 0.5% crystal violet in 25% methanol. The plates dried, and the colonies were imaged and counted with the Genesys software (Syngene). Each value reported is the mean of three biological replicates, each derived from the mean of three technical replicates.

### Decipher shRNA library screen

A library of barcoded shRNAs targeting 6316 human genes obtained from Addgene was transduced into RWPE-ERG and RWPE-KRAS cells and selected by puromycin. Cells were then subjected to six-well *trans*-well migration assays. Upon completion, trypsin was applied to the underside of the membrane to physically separate the migratory cells from the membrane. Trypsin was also applied to the inside of the chamber to separate the non-migratory cells remaining in the chamber. These two cell populations were grown separately and sequenced to determine which genes were knocked down in each population.

### RNA quantification

Total RNA was extracted from cells using the RNeasy Kit in combination with QIA-shredder columns (Qiagen). RNA was quantified via the Nanodrop 2000c (Thermo Scientific). About 1% β-mercaptoethanol was added to the RLT lysis buffer. RNA was quantified by reverse transcription and quantitative polymerase chain reaction (qPCR) as described in (24). Reverse transcription reactions contained 500 ng of RNA, 500 μM dNTPs, 100 nM oligo primers, 1× First Strand Buffer (New England BioLabs), 5 mM dithiothreitol (DTT) (Invitrogen), 40 U Murine RNase Inhibitor and 200 U Superscript III reverse transcriptase in 20 μl of total reaction volume. Reactions were incubated at 55°C for 55 min followed by 15 min at 70°C. Finally, 5 U of RNase H was added to the reaction and incubated at 37°C for 20 min. cDNA was stored at −20°C if not immediately analyzed by qPCR. Reactions for qPCR contained 2 μl of cDNA, 2 μl RNase-free water, 1× KAPA SybrFast qPCR master mix (2.6 mM MgCl_2_) and 500 nM primers in a total reaction volume of 10 μl. Two technical replicates of each sample were manually plated in 96-well plates (VWR #83009-676) and read by a RealPlex2 Mastercycler and analyzed by the Eppendorf Realplex software (Eppendorf). A standard curve was generated for each target by running five 10-fold serial dilutions of standards. The PCR program was 95°C for 3 min, followed by 40 cycles of 95°C for 15 s, 61°C for 15 s and 72°C for 30 s. Upon completion of the PCR, a melting curve was generated to validate specificity. RNA levels were normalized to 18S. The DNA oligonucleotide primers used were produced by Integrated Data Technologies. Primer specificity was screened *in silico* with UCSC’s BLAT tool. Amplicon size, primer sequences and primer locations are listed in Supplementary Table S2. Standard curve data are listed for each target in Supplementary Table S3.

### Luciferase reporter assay

RWPE cells were transiently transfected with 500 μg of ERG, mutant ERG or empty vector, and 1000 μg of renilla reporter and luciferase reporter using Trans-IT 2020. The luciferase reporter was created from a 474 bp fragment of an FHL3 enhancer (chr1:38465034–38465507, hg19) that was cloned into the firefly luciferase plasmid pGL4.25 and was described previously (23). Twenty-four hours after transfection, drug treatments were performed and cells incubated for six additional hours. Cell lysates were collected and analyzed using Promega's Dual Reporter Luciferase Assay Kit. Briefly, cells were harvested with Passive Lysis Buffer, and the lysate underwent four freeze–thaws cycles in liquid nitrogen to promote cell lysis. Luciferase Assay Reagent II was then added to the samples in a 96-well plate and the luminescence was measured. Stop and Glo was then added to quench the luciferase signal and activate the renilla. The luminescence was measured and values were normalized to renilla.

### Immunoblots

Whole cell extracts were collected with NP-40 lysis buffer [50 mM Tris–HCl, pH 7.4, 250 mM NaCl, 5 mM ethylenediaminetetraacetic acid (EDTA), 50 mM NaF and 1% Nonidet *P*-40], and then normalized by protein concentration using Bradford assays with bovine serum albumin standards. Tumor samples were diced into ∼1 mm^3^ pieces before being homogenized in 3 ml of RIPA buffer (50 mM Tris–HCl, pH 7.4, 150 mM NaCl, 1 mM EDTA, 0.1% sodium dodecyl sulfate (SDS), 1% NP-40, 0.5% sodium deoxycholate, 0.5 mM DTT) in a 40 ml capacity manual tissue grinder (Kimble #885300-0040) while on ice. Tissue extracts were then centrifuged at 14 000 rpm at 4°C to remove cell debris, and the supernatant was retained. For secreted proteins, conditioned medium was collected from the cells, and protein from the cells was separately harvested as explained above to serve as a loading control. The conditioned media was centrifuged at 500 × *g* to remove any non-adherent cells. One part of 100% trichloroacetic acid (Sigma, T0699) was added to four parts of the cleared conditioned media and incubated on ice for 30 min before centrifuging at 16 100 × *g* at 4°C for 15 min. The supernatant was discarded and the pellet was washed with 300 μl of 100% acetone (Sigma, 179124) before centrifuging at 16 100 × *g* at 4°C for 5 min. The supernatant was removed, the pellet was resuspended in SDS loading dye and the samples were boiled at 100°C for 10 min. Samples were separated on 10% SDS-polyacrylamide gel electrophoresis gels and transferred to nitrocellulose membranes (Bio-Rad) using standard procedures. Membranes were then blocked with 5% milk in TBS (10 mM Tris, pH 8.0, 150 mM NaCl), incubated with primary and secondary antibodies, and exposed to Super Signal ECL (Thermo Scientific). Antibodies used in this study are FLAG (F1804, Sigma), pS96 ERG (25), Tubulin (T9026, Sigma), TLR4 (SC-293072, Santa Cruz), EWS (SC-28327, Santa Cruz), pAKT (D9E, Cell Signaling), pMEK (9121, Cell Signaling), MEK (9122S, Cell Signaling), AKT (C73H10, Cell Signaling), HSPA8 (D12F2, Cell Signaling) and BGN (HPA003157, Atlas Antibodies).

### Co-immunoprecipitation

Magnetic Dynabeads were combined with the appropriate antibody and 250 μl of NP-40 lysis buffer (50 mM Tris–Hcl, pH 7.4, 250 mM NaCl, 5 mM EDTA, 50 mM NaF and 1% Nonidet *P*-40) in an Eppendorf tube and rotated at 4°C overnight. Cells were harvested from 15 cm plates with NP-40 lysis buffer and sonicated for two 10 s cycles using a probe sonicator. Debris was removed in a microcentrifuge at 15 000 rpm for 10 min. Protein concentration was determined by Bradford assays. Equal amount of protein was added to each tube, and 5% of each sample retained as input controls. Tubes were rotated at 4°C for 4 h. The beads were washed four times with NP-40 lysis buffer for 5 min. Samples were then resuspended in loading dye and loaded onto a gel to be run as a western described above.

### Chromatin immunoprecipitation and quantitative PCR

Chromatin immunoprecipitation (ChIP) was performed as previously described in (24). In brief, cells were cross-linked for 15 min using 1% formaldehyde (Fisher Scientific) and quenched with 2 M glycine for 5 min. The cells were then washed, lysed and sonicated (Diagenode, Bioruptor Pico) at 4°C for three cycles of 30 s on followed by 30 s off. The nuclear fraction was incubated with an ERG antibody (CM 421, Biocare) conjugated to magnetic beads (mouse Dynabeads, Thermo Fisher) for 4 h at 4°C. The beads were washed, and the DNA was isolated by a phenol/chloroform extraction. The isolated DNA was then reverse transcribed and quantified as described in (24). The DNA oligonucleotides used are listed in Supplementary Table S2.

### RNA-sequencing analysis, patient sample data, gene ontology and pathway analysis

RNA-seq data were analyzed from experiments reported in (23,25) via tophat2 and cufflinks program packages. Patient data from The Cancer Genome Atlas and the Fred Hutchinson CRC prostate cancer dataset were analyzed and visualized through cBioPortal (26–28). Correlation coefficients for *TLR4* and *ERG* expression were generated by cBioPortal. Gene ontologies for the shRNA screen were analyzed with GOrilla by uploading the top 5% of gene knockdowns that were over-represented in non-migratory ERG-positive cells (29,30). A gene list of all genes targeted by the shRNA library was uploaded as background to remove bias. Pathway enrichment analysis was performed by uploading the list of the top 5% of gene knockdowns over-represented in non-migratory ERG-positive cells compared to ERG–KRAS cells (31,32).

### Mouse xenograft tumor growth

All animal protocols described in this study were approved by the Institutional Animal Care and Use Committee at the Indiana University School of Medicine. RWPE-ERG/myristoylated AKT cells (2 × 10^6^ cells per graft) concurrent with 5 × 10^5^ mouse prostate myofibroblastic cells from Ink4A null mice (33) were subcutaneously implanted in the hind flank of male athymic nude mice using a 100 μl volume of 50:50 solution of Matrigel:RPMI medium. The mice then received intraperitoneal injections of either vehicle (DMSO) or 20 mg/kg TAK-242 three times per week, for 3 weeks. Dosage and treatment regimen were guided by a previous TAK-242 mouse study (34). The tumor size was measured by calipers and mouse mass was monitored during the treatment schedule. BrdU was injected into the animals 2 h prior to sacrifice and tumor tissues were analyzed for BrdU incorporation (immunofluorescence) as described in (35). BrdU quantification was done as described previously, expressed as a percent of BrdU-positive to total Hoescht-positive nuclei: four 20× views were quantified by NIH image and averaged for each tumor and are considered one data point, as previously determined to be optimal for tumor growth in our group (29).

## RESULTS

### Toll-like receptor 4 signaling is implicated in ERG-mediated migration

**Figure 1.**
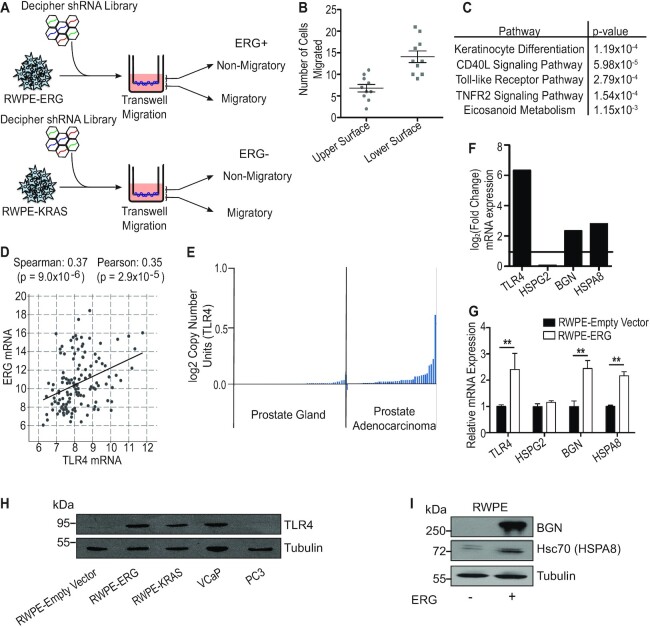
TLR4 is an important component of ERG-mediated migration. (**A**) ERG-positive (RWPE-ERG) and ERG-negative (RWPE-KRAS) prostate cells were subjected to a migration-based shRNA screen using a library from Addgene/Cellecta. (**B**) *Trans*-well migration of non-migratory and migratory cells. Cells removed from within the insert after migration were classified as non-migratory cells, and cells removed from the underside of the insert were classified as migratory. These cells were then tested in a second round of migration. (**C**) Enrichr pathway analysis output for the top 5% of over-represented genes in non-migratory ERG-positive cells. (**D**) Correlation of ERG and TLR4 mRNA expression in prostate cancer patient samples visualized via cBioPortal. (**E**) Expression of *TLR4* mRNA in normal prostate versus prostate cancer samples. (**F**) Relative mRNA levels by RNA-seq of *TLR4* and three endogenous ligands in RWPE-ERG normalized to RWPE-empty vector. (**G**) RWPE-ERG mRNA level of *TLR4* and endogenous ligands by RT-qPCR normalized to RWPE-empty vector cells. (**H**) TLR4 protein expression across a panel of ERG-positive and ERG-negative prostate cell lines. (**I**) Secreted protein expression of BGN and Hsc70 (gene name *HSPA8)* in RWPE-empty vector and RWPE-ERG conditioned media. Tubulin expression from the corresponding whole cell extracts is shown as a loading control. Shown are mean and SEM of three biological replicates and ** indicates *P*< 0.01 by Student's *t*-test.

We have previously reported that ERG can mimic KRAS in prostate cancer cells, whereby both ERG and KRAS activate a similar gene expression program that promotes cell migration (24). To determine factors that are specific to ERG function in prostate cells, we screened for genes necessary for ERG-mediated, but not KRAS-mediated prostate cell migration. We used the immortalized normal prostate cell line RWPE1 stably expressing ERG from the *HNRNP2AB1* promoter (RWPE-ERG) or overexpressing KRAS (RWPE-KRAS, also known as RWPE2). The *HNRNP2AB1* promoter is fused to ETS factors in some prostate cancer patients and this promoter results in lower, more physiological ERG expression than CMV promoter constructs, but can still drive cell migration and tumor growth (23,36). The Decipher shRNA library (Cellecta) was transformed into RWPE-ERG and RWPE-KRAS cells and each grown in a *trans*-well chamber (Figure 1A). Cells were then physically separated into two populations based on ability to migrate through the insert membrane. A second migration assay verified that separated cells retained migratory and non-migratory phenotypes (Figure 1B). These populations were sequenced to determine knockdowns enriched in non-migratory RWPE-ERG cells, but not enriched in non-migratory RWPE-KRAS cells to avoid identification of general migration factors that are not ERG-specific (Supplementary Table S4). The top 5% of over-represented hits were examined using the Enrichr pathway analysis tool, which revealed that TLR signaling was one of the top pathways enriched (Figure 1C). To further validate this finding, we subjected the same gene list to gene ontology analysis using GOrilla with the list of all genes in the shRNA library as background. GOrilla revealed multiple ontologies relating to the innate immune response, such as inflammation and defense responses, as well as response to external stimuli (Supplementary Table S5). One ontology was the response to lipopolysaccharide, which is the canonical ligand of TLR4. In our list of top enriched knockdowns, we identified several genes encoding proteins in the canonical TLR4 signaling pathway, including *TIRAP, CD14* and *RELA*.

It has been previously reported that ERG can drive TLR4 expression in prostate cells and that TLR4 signaling can contribute to an invasive phenotype in prostate cancer cells (11,22). We observed a significant positive correlation between ERG and TLR4 mRNA expression in patient tumors (Figure 1D). Increased expression of TLR4 is also apparent in prostate adenocarcinoma samples compared to normal prostate tissue (Figure 1E). Examining our previous RNA-seq data from RWPE1 cells (23), we found that ERG expression led to increased expression of *TLR4* and two non-canonical, endogenous TLR4 ligands, *HSPA8* and *BGN* (Figure 1F). A third non-canonical TLR4 ligand, *HSPG2* did not increase. These gene expression changes were verified by quantitative RT-PCR of RNA from RWPE-empty vector and RWPE-ERG cells (Figure 1G). ERG expression also drives protein expression of TLR4 in RWPE cells (Figure 1H). Secreted biglycan (*BGN*) and Hsc70 (*HSPA8)* protein levels are elevated in RWPE-ERG media compared to RWPE-empty vector (Figure 1I). Therefore, both TLR4 and some potential TLR4 ligands are upregulated when ERG is expressed, providing a possible mechanism for ERG-mediated activation of this pathway.

### TLR4 inhibitor, TAK-242, selectively inhibits ERG-mediated oncogenic phenotypes

**Figure 2.**
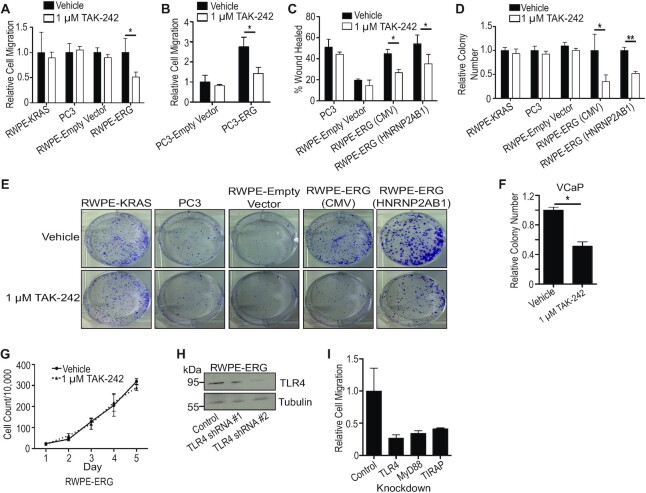
TLR4 inhibition reduces ERG’s oncogenic phenotypes. (**A**). *Trans*-well assays measure cell migration of prostate cell lines treated with 1 μM TAK-242 relative to the same line with vehicle treatment. Only the RWPE-ERG line expresses ERG. (**B**). *Trans*-well assays of PC3-empty vector and PC3-ERG cells with and without TAK-242 with migration shown relative to PC3-empty vector vehicle. (**C**). Wound-healing assay of indicated cell lines treated with DMSO or 1 μM TAK-242. Shown is percentage of the initial wound that has closed. (**D**). Number of colonies formed during low-density survival assays was normalized to indicated cell line's vehicle treatment. (**E**). Representative images of colonies from clonogenic survival assays quantified in (D). (**F**). Relative number of VCaP colonies formed during soft-agar anchorage-independent growth was normalized to vehicle treatment. (**G**). Proliferation assay of RWPE-ERG cells plated at medium density treated with TAK-242. (**H**). Immunoblot of TLR4 after shRNA-mediated knockdowns. ShRNA #2 was used in follow-up assays. (**I**). *Trans*-well migration of RWPE-ERG cells expressing shRNA knockdowns of TLR4 signaling pathway components. The number of migrated cells was normalized to the control. *N* = 2. All experiments are reported as the mean and SEM of three biological replicates unless stated otherwise. *P*-values were calculated using Student's *t*-test where * indicates *P*< 0.05 and ** indicates *P*< 0.01.

To test whether pharmacological inhibition of TLR4 signaling can specifically reduce ERG-mediated phenotypes, a specific TLR4 inhibitor, 1 μM TAK-242, was added to both ERG-positive and ERG-negative cells. Cell migration in a *trans*-well assay was compared between RWPE-ERG, RWPE-empty vector, RWPE-KRAS and the ERG-negative prostate cancer cell line PC3. TAK-242 significantly reduced cell migration in RWPE-ERG cells, but not any of the ERG-negative control cell lines (Figure 2A). The IC_50_ of TAK-242 on RWPE-ERG cell migration was calculated to be 1.1 μM, whereas the IC_50_ in the ERG-negative PC3 cells was more than 4-fold higher, at 4.8 μM (Supplementary Figure S1). Exogenous expression of ERG in PC3 cells caused a 3-fold increase in cell migration that was abrogated by TAK-242 (Figure 2B). Wound-healing assays provided an alternative measure of migration. TAK-242 had no effect on wound closure in ERG-negative lines PC3 and RWPE-empty vector, but reduced migration in RWPE cells expressing ERG from both strong (CMV) and weak (HNRNP2AB1) promoters (Figure 2C). This trend of selectively inhibiting ERG-positive cells extended into clonogenic survival assays (Figure 2D and E). The only prostate cancer cell line with known ERG dependency is the androgen-dependent cell line VCaP. We found VCaP cells unable to grow in conditions necessary for migration or clonogenic survival assays, so a soft-agar growth assay was used. TAK-242 significantly inhibited anchorage-independent growth of VCaP cells (Figure 2F). Consistent with previous findings that ERG can promote cell migration, clonogenic survival and tumor growth (2–4,23,25), but not standard high-density proliferation of prostate cells, TAK-242 had no effect on standard 2D proliferation of RWPE-ERG cells (Figure 2G). This is consistent with ERG regulating processes that are important for transformation, but not standard proliferation and indicates that TAK-242 inhibits known ERG-mediated phenotypes. To confirm TAK-242 was functioning through the inhibition of TLR4 and not off-targets, RWPE-ERG cells were subjected to shRNA knockdowns of TLR4 (Figure 2H) as well as TIRAP and MyD88, two downstream adapter proteins in the TLR4 pathway. These TLR4 pathway knockdowns displayed reduced RWPE-ERG migration (Figure 2I) comparable to TAK-242 treatment. These data suggest the observed phenotypic changes after drug treatment are due to alterations in the drug's primary target, TLR4.

### Phosphomimetic ERG mutant is resistant to TAK-242

**Figure 3.**
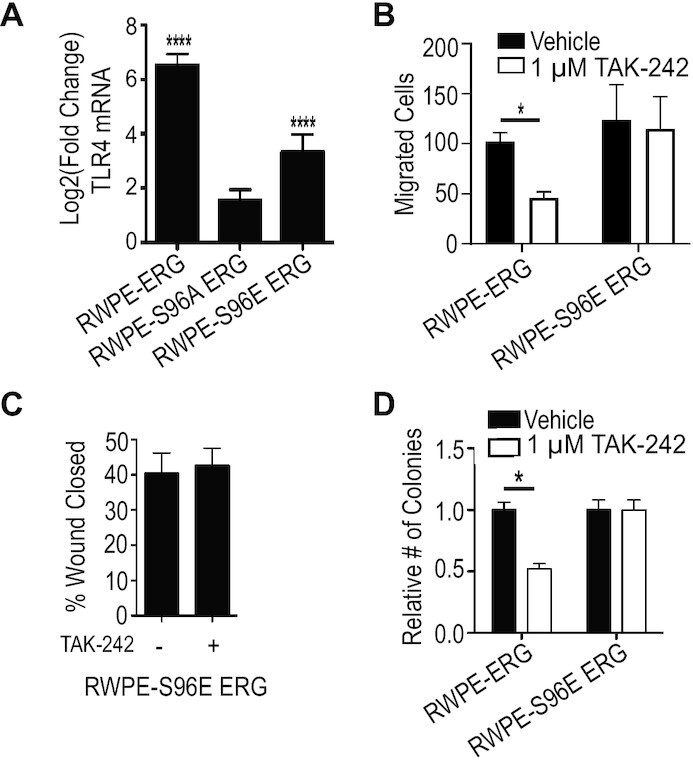
ERG phosphomimetic is resistant to TLR4 inhibition. (**A**) Relative mRNA levels by RNA-seq of *TLR4* in RWPE-ERG, RWPE-S96A ERG and RWPE-S96E ERG normalized to RWPE-empty vector. (**B**) Number of cells of indicted cell types per field of view migrated through a *trans*-well with TAK-242 or vehicle treatment. (**C**). Wound-healing assays measured migration of RWPE-S96E ERG with and without TAK-242 (1 μM). (**D**) Clonogenic survival of RWPE-ERG and RWPE-S96E ERG with and without TAK-242. All experiments are the mean and SEM of three biological replicates; * indicates *P*< 0.05 and **** indicates *P*< 0.0001 determined by Student's *t*-test.

Increased expression of TLR4 and its ligands by ERG supports the previously reported model that ERG is functioning upstream of TLR4 (22). However, TLR4 signaling can activate multiple signaling pathways that are important for ERG function such as the PI3K/AKT pathway and MAPK pathway (37–40,33). This suggests the possibility of TLR4 functioning upstream of ERG. To distinguish between these two potential mechanisms by which TLR4 inhibition alters ERG-mediated phenotypes, we tested a phosphomimetic mutant of ERG in which serine 96 is mutated to a glutamic acid (S96E). We have previously reported that ERG S96E can activate transcription and ERG-mediated phenotypes as well as, or better than, wild-type ERG, while the phosphonull, ERG S96A, does not promote transcriptional activation, or any ERG-mediated phenotype (25). Further, analyzing previous RNA-seq data, we find that ERG S96E can activate TLR4, while ERG S96A cannot (Figure 3A). When RWPE cells expressing S96E ERG were treated with TAK-242 and subjected to the *trans*-well migration assay, the reduction in migration observed with RWPE-ERG cells was lost (Figure 3B). TLR4 inhibition also failed to alter migration of RWPE-S96E ERG cells measured by wound healing (Figure 3C). The resistance to TAK-242 by phosphomimetic ERG also extended to clonogenic survival (Figure 3D). These data suggest that the critical role of TLR4 signaling in ERG function is not downstream of ERG, but is upstream, where it can be bypassed by a constitutively active ERG mutation.

### TAK-242 functions through disruption of MAPK pathway independently of PI3K/AKT signaling

**Figure 4.**
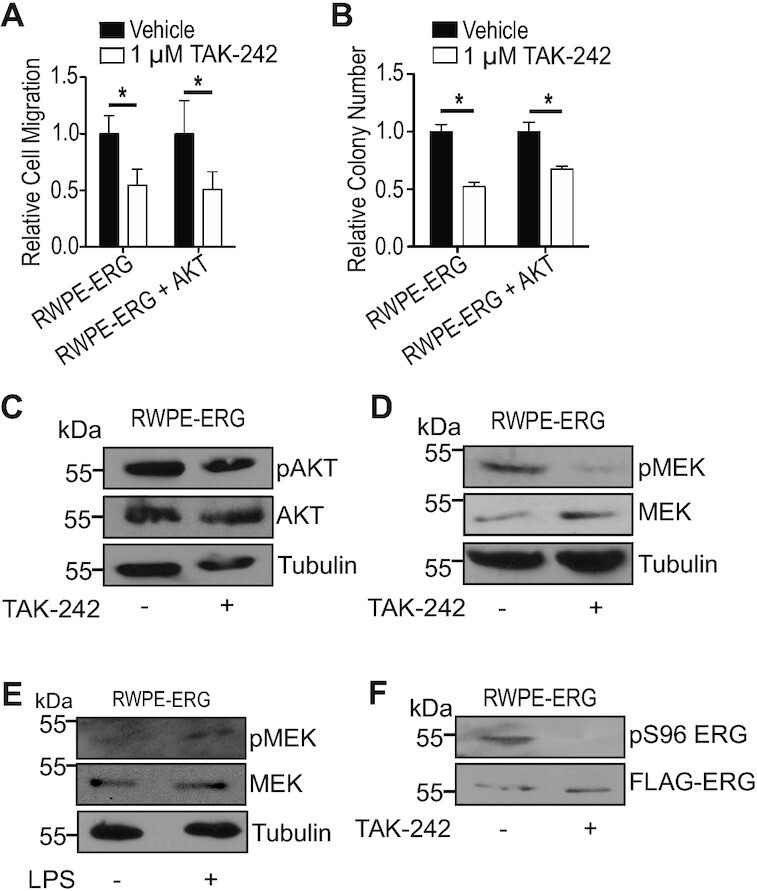
TLR4 regulates ERG through MAPK pathway and not PI3K/AKT pathway. (**A**) *Trans*-well migration of RWPE cells expressing either ERG or myristoylated AKT and ERG (RWPE-ERG + AKT) treated with TAK-242 shown relative to vehicle. (**B**) Clonogenic survival of RWPE-ERG and RWPE-ERG + AKT treated with 1 μM TAK-242 shown relative to vehicle. (**C**) Immunoblot of phosphorylated AKT in RWPE-ERG cells treated with 1 μM TAK-242. Immunoblot of phosphorylated MEK in RWPE-ERG cells treated with TAK-242 (**D**) or 0.5 μg/ml lipopolysaccharide (**E**). (**F**) Immunoblot of phosphorylated ERG at residue serine 96 in RWPE-ERG cells treated with 1 μM TAK-242. Experiments in (A) and (B) are the mean and SEM of three biological replicates, and * indicates *P*< 0.05 determined by Student's *t*-test.

To determine specific factors and pathways that mechanistically connect TLR4 to the activation of ERG, we first considered the PI3K/AKT pathway, as it can be activated by TLR4 and is important for ERG function (4). To determine whether TLR4 activates ERG via PI3K/AKT signaling, functional assays were performed with a cell line that expresses ERG as well as constitutively-active myristoylated AKT. Despite having constitutively active AKT, TAK-242 inhibited migration and clonogenic survival similar to cells expressing ERG alone, suggesting TAK-242 does not function through this pathway (Figure 4A and B). TLR4 inhibition also had no effect on pAKT levels (Figure 4C), further indicating that TLR4 is activating ERG independently of PI3K/AKT signaling.

The RAS–RAF–MEK–ERK (MAPK) pathway can also be activated through TLR4 signaling. This pathway activates ERG through phosphorylation of serine 96 (25). Upon TAK-242 treatment, there was reduced phosphorylation of MEK (Figure 4D), indicating reduced activity of this kinase. When lipopolysaccharide, the canonical ligand of TLR4, is added to RWPE-ERG cells, phosphorylation of MEK increased (Figure 4E). Further, phosphorylation of ERG at serine 96 was reduced upon TAK-242 treatment (Figure 4F). These data suggest TLR4 inhibition reduces ERG’s activity by downregulating the MAPK pathway, thus preventing the activating phosphorylation at serine 96.

### TLR4 inhibition reduces ERG’s ability to transcriptionally activate its targets

**Figure 5.**
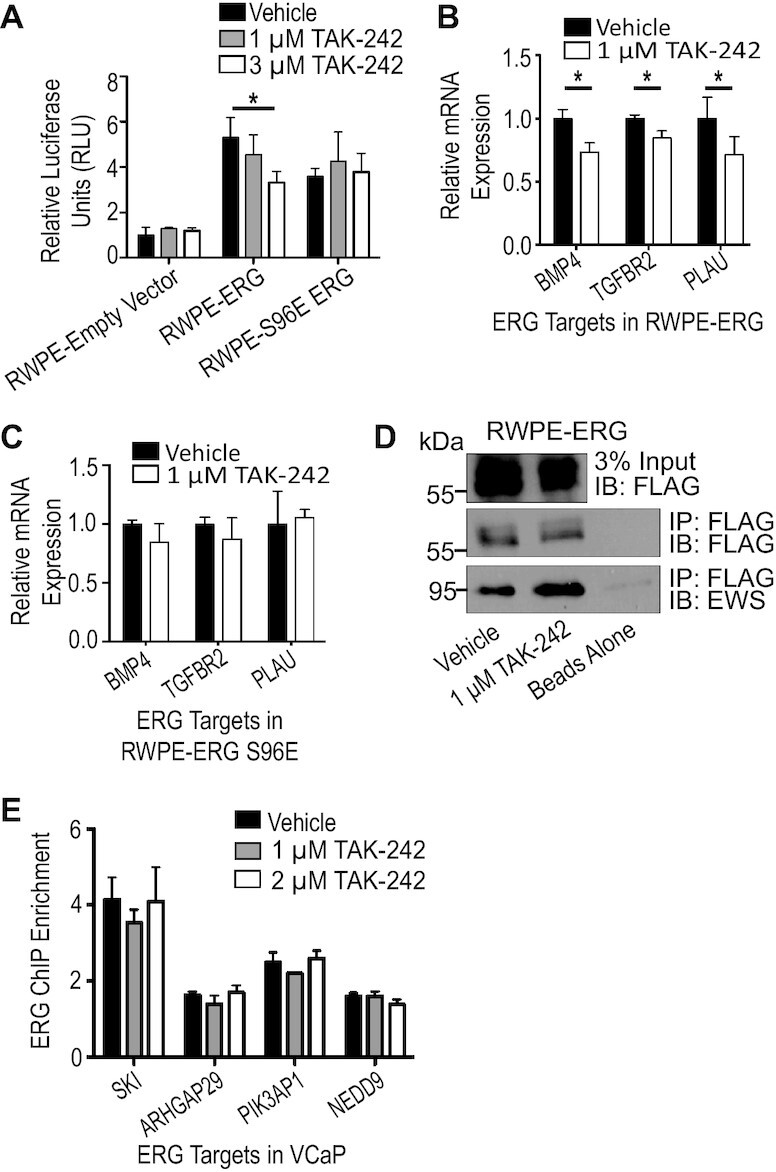
TLR4 inhibition reduces ERG’s ability to transcriptionally activate its targets. (**A**) Luciferase assay using an ERG reporter containing the FHL3 enhancer compares RWPE-empty vector and RWPE-ERG treated with vehicle or 3 μM TAK-242. (**B**) Expression of three ERG target genes identified previously (23) in RWPE-ERG measured by RT-qPCR with or without 1 μM TAK-242. Expression was first normalized to *18S* and then normalized to vehicle treatment. (**C**) Expression of three ERG target genes in RWPE-S96E ERG as in (B). (**D**) Immunoblot of co-immunoprecipitation of ERG and EWS during TAK-242 treatment using FLAG beads. (**E**) ChIP qPCR of ERG targets identified previously (23), with and without TAK-242. ChIP enrichment is the copy number of the target region normalized to two control genomic regions. All experiments are mean and SEM of three biological replicates. *P*-values were determined by Student's *t*-test, and * indicates *P*< 0.05.

To determine which specific molecular functions of ERG are altered by TLR4 inhibition, luciferase assays were performed with a reporter containing the *FHL3* enhancer, which we have previously shown is activated in an ERG-dependent manner (23). Addition of ERG to RWPE cells caused a 5-fold increase in signal (Figure 5A) and treatment with 3 μM TAK-242 significantly reduced this activity. This suggests that ERG’s ability to transcriptionally activate its targets is impaired by TLR4 inhibition. To further confirm this observation, RT-qPCR measured expression of direct ERG target genes identified from ChIP-seq and RNA-seq data (23,41). Treatment with TAK-242 resulted in significant decreases in mRNA of each of these ERG targets (Figure 5B). In contrast, there was no significant decrease in expression of these genes in cells expressing ERG-S96E and treated with TAK-242 (Figure 5C). This is additional confirmation that TLR4 is promoting ERG activation through the serine 96 residue. TAK-242 did not alter interaction of ERG with the co-activator EWS (Figure 5D) or alter ERG binding to the genome (Figure 5E), consistent with our previous findings that S96 phosphorylation does not regulate these processes (25).

### TAK-242 reduces tumor growth in ERG-positive mouse xenografts

**Figure 6.**
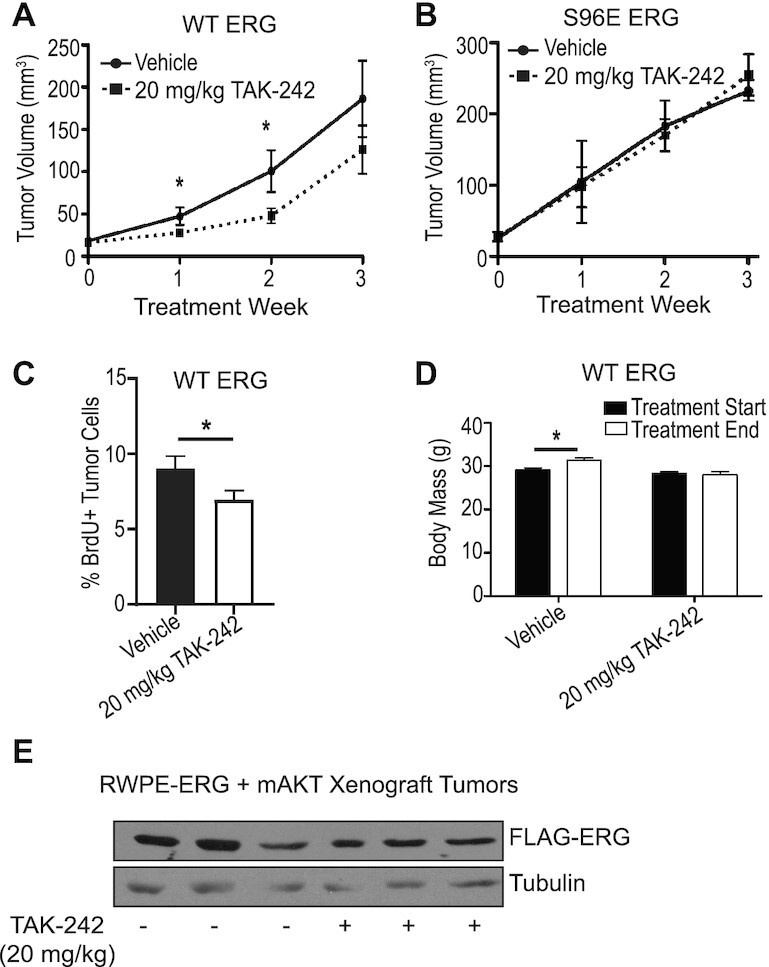
TLR4 inhibition reduces ERG-positive tumor growth in mice. (**A**) Mouse flank xenograft tumor growth measured by caliper. RWPE-ERG-myristoylated AKT cells were co-injected with fibroblasts. Tumors were allowed to grow to 20 mm^3^,and then treated with vehicle (0.1% DMSO) or 20 mg/kg/day TAK-242. Mice received intraperitoneal injections daily for 3 weeks. *N*= 11 vehicle-treated mice and *N*= 12 TAK-242-treated mice. (**B**) As in (A), but using RWPE-ERG S96E. *N* = 3 vehicle-treated mice and *N*= 4 TAK-242-treated mice. (**C**) Mice shown in A were treated with BrdU 2 h prior to sacrifice, and BrdU staining was detected by immunofluorescence. *N* = 6. (**D**) Total mouse body mass before and after 3 weeks of drug treatment. (**E**) Expression of exogenously expressed FLAG-tagged ERG in tumor samples taken from three vehicle-treated and three TAK-242-treated mice. The reported values are averages of biological replicates reported above with SEM. *P*-values were determined by Fisher's exact *t*-test, and * indicates *P*< 0.05.

One function of ERG in prostate cells, particularly when coupled with PI3K-AKT pathway activation, is the promotion of tumor growth (4,23). We have previously shown that RWPE cells will grow as xenograft tumors when expressing both ERG and myristoylated AKT and when combined with reactive stromal cells of a myofibroblastic phenotype (23). In this experiment, the RWPE cells were co-injected with mouse prostate myofibroblastic cells that were harvested from Ink4A null mice as previously described (42). To test whether TLR4 inhibition can alter ERG function *in vivo*, we treated these xenografts with TAK-242. When tumors reached 20 mm^3^, the mice were treated intraperitoneally with 20 mg/kg TAK-242 three times weekly for 3 weeks. TAK-242 reduced tumor volume by approximately half in the first and second weeks of treatment (Figure 6A). These data suggest that TAK-242 can reduce ERG-dependent tumor growth. Interestingly, tumors made with the phosphomimetic S96E ERG mutant grew faster than those expressing wild-type ERG, and showed no responsiveness to TAK-242 (Figure 6B). In addition, TAK-242 treatment *in vivo* was able to reduce BrdU incorporation (29) in ERG-AKT tumors (Figure 6C and Supplementary Figure S2), indicating that proliferation rate is a primary mechanism of the reduced tumor size elicited by TAK-242. Of note, mice treated with TAK-242 did fail to gain the slight increase in body mass that vehicle-treated animals gained across the 3 weeks of treatment, indicating that there could be some systemic effect associated with this treatment schema (Figure 6D). Expression of exogenously expressed FLAG-tagged ERG was found to be similar in tumor samples from three mice that received vehicle treatment and three mice that received TAK-242 (Figure 6E). All told, Figure 6 represents an *in vivo* confirmation of the ERG functional inhibitor effects elicited by TAK-242 in *in vitro* studies.

## DISCUSSION

Our findings indicate that TLR4 signaling can activate ERG transcriptional function in prostate cells via the RAS/MAPK pathway and that pharmacological inhibition of TLR4 can reduce ERG-mediated phenotypes including tumor growth. Consistent with literature reports (22), we found that ERG can increase *TLR4* expression. Further, we find that ERG can activate two endogenous TLR4 ligands, *BGN* and *HSPA8*, indicating a potential positive feedback loop. However, experiments with the TLR4 inhibitor TAK-242 and a constitutively active ERG mutant (S96E) indicate that downstream activation of TLR4 signaling is not necessary for ERG-mediated phenotypes. Instead, TLR4 signaling is necessary to promote ERG phosphorylation, which allows full transcriptional activation of target genes.

A previous report indicated that knockdown of TLR4 in PC3 prostate cancer cells can reduce invasion and proliferation (11). We have observed that ERG-positive cells express much higher levels of TLR4 than ERG-negative cells such as PC3, and ERG-positive cell lines are more sensitive to TLR4 inhibition. This indicates that while TLR4 signaling may play some role in ERG-negative prostate cells, ERG-positive cells are more dependent on active TLR4 signaling to maintain activation of ERG.

The TLR4 signaling pathway is an immune pathway that allows cells to respond to bacterial infections by sensing LPS on bacterial cell walls. TAK-242 was developed as a treatment for sepsis, with a goal of tempering the immune response to infection (43–46). The *TMPRSS2/ERG* gene rearrangement found in prostate tumors results in ERG expression in prostate cells, a cell type where ERG is not normally expressed. ERG is normally expressed in blood vessels and immune cells (47–50). It is possible that aberrant expression of ERG in prostate cells due to *TMPRSS2/ERG* promotes immune cell-specific gene expression programs, including a program that allows TLR4 signaling. However, our data indicate that the critical role of TLR4 signaling in prostate cells is upstream of ERG, where TLR4 promotes activation of the MAPK pathway and subsequent ERG S96 phosphorylation. The ability of ERG to increase expression of TLR4 and TLR4 endogenous ligands has the potential to create a positive feedback loop. Therefore, ERG and TLR4 activity is self-reinforcing and could mediate a stable cell fate change when expressed in prostate cells.

Future work will be important to examine how TLR4 inhibition affects ERG-positive prostate cancer in animals with an intact immune system and possible synergies with other treatments. As TLR4 signaling is an immune-response pathway, it is likely to interplay with the role of the immune system in cancer, an aspect that we did not test in our immune-deficient model. While acute TLR4 activity is known to drive pro-inflammatory cytokine production, evidence is emerging that prolonged TLR4 signaling can contribute to a more immunosuppressive response, leading to immune escape by cancer cells (16,51–52). TLR4 inhibition in immune-competent individuals could not only reduce ERG function but also relieve suppression of the immune system in the tumor. There could also be synergy with other treatments. In particular, taxanes are a widely used chemotherapeutic for metastatic prostate cancer. Interestingly, paclitaxel has been reported to activate the TLR4 pathway, and this activation can promote resistance (15,53). There is also a report that paclitaxel can promote metastases in some cancers through TLR4 signaling (13). Therefore, it is possible that treatment of ERG-positive prostate cancer with TLR4 inhibitors could simultaneously inhibit ERG function and decrease resistance to chemotherapy.

## Supplementary Material

zcaa046_Supplemental_File

## Data Availability

Quantitative PCR complies with MIQE Guidelines. See Supplementary Tables S2 and S3.
