# Supplementary material for: Toll-like receptor 4 signaling activates ERG function in prostate cancer and provides a therapeutic target
Source: NAR Cancer. 2021 Jan 27;3(1):zcaa046. doi: 10.1093/narcan/zcaa046 (PMC7848947; doi:10.1093/narcan/zcaa046)
Supplement: zcaa046_Supplemental_File [file zcaa046_supplemental_file.pdf]

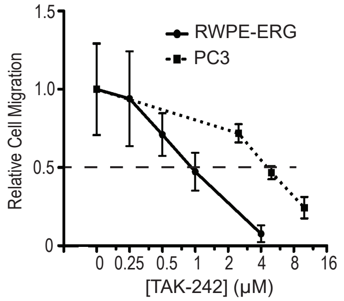

Figure S1. ERG-positive prostate cell are more sensitive to TLR4 inhibition than ERG-negative prostate cells. RWPE-ERG and PC3 cells were treated with a range of TAK-242 concentrations and subjected to trans-well migration assays.

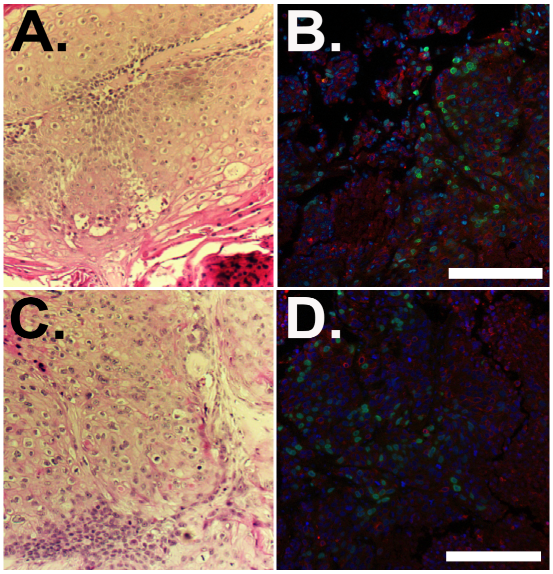

Figure S2. Representative H&E and BrdU staining stained views of RWPE-ERG + mAKT tumors. Tumors were treated with vehicle **(A,B)** or TAK-242 (**C,D)** as described in Figure 6, where tabulated data are shown. Scale bar represents 100 µm; BrdU labeled cells **(B,D)** are in green, while pan-cytokeratin staining is shown in red to demonstrate epithelial nature of the tumors.

Supplementary Table S1. shRNA Primer sequences.

Target	Sequence
TLR4 shRNA #1 Forward	CCGGAAGGTGATTGTTGTGGTGTCCCTCGAGGGACACCACAACAATCACCTTTTTTTG
TLR4 shRNA #1 Reverse	AATTCAAAAAAAGGTGATTGTTGTGGTGTCCCTCGAGGGACACCACAACAATCACCTT
TLR4 shRNA #2 Forward	CCGGAACTTGTATTCAAGGTCTGGCCTCGAGGCCAGACCTTGAATACAAGTTTTTTT
TLR4 shRNA #2 Reverse	AATTCAAAAAAACTTGTATTCAAGGTCTGGCCTCGAGGCCAGACCTTGAATACAAGT
MyD88 shRNA Forward	CCGGGCCTGTCTCTGTTCTTGAACTCGAGTTCAAGAACAGAGACAGGCTTTTTG
MyD88 shRNA Reverse	AATTCAAAAAGCCTGTCTCTGTTCTTGAACTCGAGTTCAAGAACAGAGACAGGC
TIRAP shRNA Forward	CCGGAAGTCAAAGAAGCTGTCATGCCTCGAGGCATGACAGCTTCTTTGACTTTTTTT
TIRAP shRNA Reverse	AATTCAAAAAAAGTCAAAGAAGCTGTCATGCCTCGAGGCATGACAGCTTCTTTGACT

Supplementary Table S2. qPCR Target Data.

Target	Sequence	Amplicon Size	Primer Location	Splice Variants Targeted	Target Sequence Accession Number
18S Forward	GGTGAAATTCTTGGACCGGC	196 bp	Exon 1	-	NR_145820.1
18S Reverse	GACTTTGGTTTCCCGGAAGC	196 bp	Exon 1	-	NR_145820.1
TLR4 Forward	TCTCAACCAAGAACCTGGACC	262 bp	Exon 3	Variant 1/3	NM_003266.4
TLR4 Reverse	AGAGATGCTAGATTTGTCTCC	262 bp	Exon 4	Variant 1/3	NM_003266.4
BGN Forward	CGATGGGCCATTCATGATGA	186 bp	Exon 2	Variant 1	NM_001711.6
BGN Reverse	GTGTCAGGGGAGATCTCTTT	186 bp	Exon 3	Variant 1	NM_001711.6
HSPA8 Forward	CCAACACAGTTTTTGATGCC	183 bp	Exon 2	Variants 1-2	NM_006597.6
HSPA8 Reverse	TCAGAACCATAGAAGACACC	183 bp	Exon 3	Variants 1-2	NM_006597.6
HSPG2 Forward	CCAGATGGTTTATTTCCGAGCC	138 bp	Exon 2/3 Junction	Variants 1-2	NM_001291860_0
HSPG2 Reverse	TTCAAGTACTCCGACTCCAG	138 bp	Exon 5	Variants 1-2	NM_001291860_0
BMP4 Forward	TACATGCGGGATCTTTACCGG	227 bp	Exon 3	Variants 1-9	NM_001347916.1

BMP4 Reverse	TCTGCAGAGGAGATCACCTCG	227 bp	Exon 4	Variants 1-9	NM_001347916.1
TGFBR2 Forward	GGAAGTCTGTGTGGCTGTATG	188 bp	Exon 3	Variants 1-2	NM_003242.6
TGFBR2 Reverse	TGCACTCATCAGAGCTACAGG	188 bp	Exon 4	Variants 1-2	NM_003242.6
PLAU Forward	GCTGACACGCTTGCTCACC	118 bp	Exon 8	Variants 1-3	NM_001145031.2
PLAU Reverse	CGTTATACATCGAGGGCAGGC	118 bp	Exon 9	Variants 1-3	NM_001145031.2
SKI Forward	GGAGCCAGTGACCTCATTTC	106 bp	Intron 1	-	NM_003036.4
SKI Reverse	GCACAGTGTGTTGCATACAGG	106 bp	Intron 1	-	NM_003036.4
ARHGAP29 Forward	GGAGTCCTGTAAGGGGAGGA	192 bp	Intron 1	-	NM_001328664.2
ARHGAP29 Reverse	CGGCTAGTTGCCGTCCAG	192 bp	Intron 1	-	NM_001328664.2
PIK3AP1 Forward	GTGTGTGAGCACCAGCTGTC	198 bp	Enhancer	-	ENST00000339364.10
PIK3AP1 Reverse	TCAGGAGACCAGATCCAAGG	198 bp	Enhancer	-	ENST00000339364.10
NEDD9 Forward	AGAGACATGGTTTCGCCATC	190 bp	Intron 2	-	ENST00000504387.5
NEDD9 Reverse	CTTTGCATTAGGGCAGGAAG	190 bp	Intron 2	-	ENST00000504387.5

Supplementary Table S3. qRT-PCR standard curve data.

Figure 1G

Target	18S	HSPG2	BGN	18S	HSPA8	TLR4
Slope	-2.92	-3.33	-3.29	-2.95	-3.36	-3.70
Y-Intercept	31.04	35.57	39.72	31.68	37.21	40.34
Efficiency	1.20	1.00	1.01	1.18	0.98	0.86
R^2	0.99	1.00	1.00	0.99	1.00	0.97
LDR	10^6 - 10^{10}	10^3 - 10^7	10^3 - 10^7	10^6 - 10^{10}	10^3 - 10^7	10^3 - 10^7

Figure 5B

Target	18S	BMP4	TGFBR2	18S	PLAU
Slope	-2.96	-3.36	-3.40	-3.07	-3.34
Y-Intercept	31.72	38.42	39.13	32.70	37.80
Efficiency	1.18	0.99	0.97	1.12	0.99
R^2	0.99	1.00	1.00	0.98	1.00
LDR	10^6 - 10^{10}	10^3 - 10^7	10^3 - 10^7	10^6 - 10^{10}	10^3 - 10^7

Figure 5C

Target	18S	BMP4	TGFBR2	18S	PLAU
Slope	-3.02	-3.34	-3.46	-2.93	-3.27
Y-Intercept	32.59	39.40	40.57	31.15	41.29
Efficiency	1.14	0.99	0.95	1.20	1.02
R^2	0.99	1.00	1.00	0.99	0.99
LDR	10^6 - 10^{10}	10^3 - 10^7	10^3 - 10^7	10^6 - 10^{10}	10^3 - 10^7

Figure 5E

Target	Negative Control #1	Negative Control #2	SKI	ARHGAP29	PIK3AP1
Slope	-3.60	-3.50	-2.99	-4.04	-3.12
Y-Intercept	36.11	35.08	34.20	38.53	35.22
Efficiency	0.90	0.93	1.16	0.77	1.09
R^2	1.00	1.00	0.99	0.98	1.00
LDR	10^1 - 10^4	10^1 - 10^4	10^1 - 10^4	10^1 - 10^4	10^1 - 10^4

Target	Negative Control #1	Negative Control #2	SKI	ARHGAP29	PIK3AP1	NEDD9
Slope	-3.35	-3.11	-3.16	-3.58	-3.53	-3.20
Y-Intercept	34.88	34.07	34.46	37.12	36.65	33.14
Efficiency	0.99	1.10	1.07	0.90	0.92	1.06
R^2	0.97	0.99	0.99	1.00	0.99	0.99
LDR	10^1 - 10^4	10^1 - 10^4	10^1 - 10^4	10^1 - 10^4	10^1 - 10^4	10^1 - 10^4

Target	Negative Control #1	Negative Control #2	SKI	ARHGAP29	PIK3AP1	NEDD9	PIK3AP1	NEDD9
Slope	-3.11	-3.38	-2.95	-3.06	-4.00	-3.48	-3.75	-3.55
Y-Intercept	33.69	34.99	34.10	35.23	37.99	34.52	37.01	33.94
Efficiency	1.10	0.98	1.18	1.12	0.78	0.94	0.85	0.91
R^2	1.00	1.00	1.00	0.99	0.96	0.99	1.00	0.99
LDR	10^1 - 10^4	10^1 - 10^4	10^1 - 10^4	10^1 - 10^4	10^1 - 10^4	10^1 - 10^4	10^1 - 10^4	10^1 - 10^4

Target	Negative Control #1	Negative Control #2	SKI	ARHGAP29
Slope	-3.39	-3.23	-3.33	-3.58
Y-Intercept	34.75	34.14	34.75	36.71
Efficiency	0.97	1.04	1.00	0.90
R^2	0.99	1.00	1.00	0.99
LDR	10^1 - 10^4	10^1 - 10^4	10^1 - 10^4	10^1 - 10^4

Supplementary Table S4. Top 5 percent of genes identified by shRNA migration screen.

Positive regulators of cell migration using top 5 percentile	RWPE-ERG specific	common	RWPE-KRAS specific
	COL3A1	GUCA1A	FANCM
	PARP2	FDPS	FIGF
	C1QB	MAPK7	PLTP
	TEP1	COL24A1	MAP2K2
	CDK9	ABCB1	FPGS
	TIRAP	EXT1	PPP2CA
	CD47	FYN	MBD1
	THBD	ADA	DDX52
	RPS6KB1	HBXIP	CDC34
	CACNA1F	HLA-DQA2	EEA1
	SALL4	PLCD1	INPP1
	GRB7	FGF8	CCNC
	PTGER4	B3GAT2	SELPLG
	DISC1	STX1A	PTCH1
	NEU3	CAV2	EXTL3
	BUB3	SMARCC2	WASL
	GGCX	CYP3A43	CR1
	CHKB	GTF3C3	GRWD1
	QPCT	ATP6V1C2	GGA1
	SULT1A3	RALBP1	ADCYAP1R1
	EED	EIF4E	CHIA
	MPST	UBE2D2	GADD45G
	FSHB	PTK6	IL17RB
	DSE	GDNF	HLA-DOB
	RAD50	RAD51L1	ADORA2B
	PDE7A	RCAN1	BCL2L2
	SOCS7	UBE3A	SMURF1
	ATF1	ESCO1	BTC
	RAB24	APOC2	GNAI3
	TNNT1	CCR4	DOCK1
	PDHA2	ZMAT3	FBXW7
	LRDD	EIF2AK3	GCDH
	VCAN	NCF2	CFI
	PANK1	HLCS	ABCG1
	ATP5G1	VIM	SH3GL2
	PDXP	HMGCS2	TRH
	TLX3	TRAF5	MERTK
	TPMT	ITCH	CHST1
	HGF	DPAGT1	TNKS2
	NFKBIL2	INSR	DNMBP

BAP1	LILRB3	CSAD
CYGB	RB1	CD38
AK3	PSMB1	AHR
EHMT1	B3GNT3	XRCC1
PIGT	WASF2	HSPA1L
PPP3R1	MYEF2	SKIL
SPRY4	XDH	LPAR2
IL13RA2	SUZ12	PTGER1
SUMO3	POU5F1	PSMD2
PAFAH1B2	DDC	COASY
TUBB3	TIMP2	IFNA4
IL22RA2	EFNA2	PLA2G7
SORBS1	ME2	PIP3-E
ATP5I	PSMD14	CDH5
PTGES	MAPK8	MTHFR
WWP1	ATP5D	CASP8AP2
HEY1	C4B	PALB2
HSF1	ATP1A3	THBS2
TRPM7	CDKN1B	LRRC15
GNAL	PPCS	ATP5B
TEK	TGFBR1	IL11RA
MTA2	CEP290	OBFC1
VHL	CFHR3	GPX6
CXCL10	PSMD4	GPR18
RNF4	KRT17	CALCRL
OPRM1	BLNK	RPL12
IL17F	PIK4CA	PUM1
BCL2A1	PRKCZ	PIK3C2G
APBB1	HTR1B	CD34
SCYL1	ABCA4	MCM3
DVL3	GOT1	POLR3H
ATP6V1G1	MCM5	TXNRD2
RGS1	MAPK1	PRL
CAPNS1	PDCD1	CD53
ACACA	GDF1	CD3D
FBP1	DNAJA3	CFD
GTF2E2	LHPP	EZH2
HUS1	MYH8	FGF4
RELA	MGAT1	LOC283398
IL9R	VKORC1	HMGA1
HSD17B6	NUDC	ATG4A

BMP4	GALNT7	GLA
ABCA9	CBR1	IL21R
GSR	IL15	GABRD
NEUROD1	UGT1A8	PAFAH1B3
DIO2	CCND3	POLB
S100A4	WNK1	MCM4
RGS4	EP300	SMCHD1
C1QL4	TP53	LEFTY2
PPME1	PTGDS	CA8
MAP3K1	PDHB	EPHB2
MAPKAPK2	IPMK	SULT1A1
PRKCG	GDF10	FABP7
POLK	PGAM1	SLC22A6
CD3EAP	KRT7	BBS2
GPD2	IL27	ANGPT2
NFATC2	VDR	CITED2
CELA3B	TTF2	SRXN1
CD14	GBF1	MEFV
ATP2B1	PSMD13	ABCA2
TNFAIP3	BYSL	MUC5AC
DPP4	PDE6H	NPPA
SDC2	CSNK1D	RNF19A
VTN	MADD	HYAL2
FBL	NCAM1	ARHGAP1
NFYA	PDE7B	UBE2L3
GLI4	LAP3	TUBA1A
ANXA6	G3BP2	PROC
AACS	KLRC1	GYG1
NR1D2	FBP2	GSTO2
P2RY6	KLK10	NR1I2
CDC40	EMR2	KLK2
NDUFB8	PSMF1	MTHFD2
C1QC	EIF4EBP1	UGCG
NCOA4	UBE2E3	CD276
GRM4	COL5A3	F9
RAB11A	COL27A1	CTNND1
PPAP2B	FOXO3	SERPINA5
NANOG	CCL11	C8A
PLCE1	MEF2D	PPP2R2C
UBE2M	PSMB7	PSMA3
TERF2IP	ADH1C	DPEP1

IFNA16	STXBP1	SNAP47
SIRPA	RELB	HIST1H2AA
APOBEC3G	ITGB1BP3	MARCO
KLKB1	GK2	CPSF3
GLI3	CYP2A6	CDA
LMO2	MAPK9	ATP5J2
GHRHR	NT5C1A	SUV39H1
ABCB11	TAF1L	SUZ12
PRKCQ	GSTM2	EIF2AK3
ANK2	PINX1	CLEC10A
BMPR2	XRCC4	NFKB1
ITPR3	ACVRL1	CHUK
ABCG2	DGKI	GPR183
SNX6	ADORA1	SULT1E1
KDELR2	FOXA2	TPP1
CHDH	MAP2K5	UGT1A8
MEPE	ABCC2	CCNA1
PRIM1	NFATC4	ABCC5
MAP3K3	HK1	RASA1
IL6ST	BBC3	HES6
CD8B	PPP1R13B	ASRGL1
EPPK1	PTTG1	SRP68
MAP3K5	ACVR1C	DGKQ
EBF1	FAM59A	ATG2A
DCLRE1A	PDPK1	SMAD4
RAD18	ACP6	ALG10B
CPSF4	GNA11	FECH
IFT57	AMDHD1	LDHAL6B
ZFYVE9	DGKA	NEFH
PLA2G4D	ADAM9	LOC283412
DAG1	SYNJ2	LIG4
CELSR2	COX5A	BZW1
EIF4EBP2	CCL22	PPCS
BCL9	CR2	EFNB1
CLYBL	SLC30A8	CD207
AK7	MMRN1	IREB2
ARAF	DPYSL5	UBE2E1
RAB1A	OGDH	PIK3R5
CDK5R1	CHPT1	PDGFB
SMARCD1	PIGH	PTGFR
COL17A1	NARS	IMMT

GCA
STAG2
PON2
BDKRB2
GLI2
PTGIS
ADH6
TNIP3
RAD9A
NRIP1
POLR3B
HSD17B2
STAT6
GNMT
GALNS
IDS
ATP5H
ATP5F1
USP9X

MOG
NDUFA5
GPX1
COQ2
SOX6
ATF4
SLC25A1
BMP15
CYP11A1
FLNC
RENBP
FST
KRT7
GDI1
NRG1
CD40
CAMK4
SLC18A3
RALBP1
PIK4CA
POLD3
VKORC1
GRM5
ARHGDIB
CBX4
NEDD8
PFKP
GOT1
SRP72
CTCF
HS3ST3B1
CD200R1
DHH
SLC6A3
ATP6V1F
WASF2
CTTN
PSMD6
CD86
ARHGAP5
CXCR3

AFP
PGA5
GNPDA2
LCT
IL7
DDC
VWF
CDKN1B
IL27
PORCN
DPAGT1
SLC12A1
STAT3
GAL3ST1
RPSA
CSF1R
DBN1
TCERG1
BAG5
PIK3CB
MCC
E2F4

DARS
GPR68
SNW1

RWPE-ERG specific	common	RWPE-KRAS specific	**Negative regulators of cell migration** using top 5 percentile
DDX52	BNIP1	RBP4	
DDO	ALG13	IFI44	
NUMA1	F11	CHP	
POLR1D	APOBEC3C	C9ORF47	
EGLN3	NDUFB4	GPD2	
COL21A1	CD96	FZD5	
MTIF2	PRC1	DDX50	
SSTR1	PC	GRIN2A	
HDC	ACTN1	DUSP14	
NDUFS7	ACAA2	PAK3	
ATOH1	HRK	AZIN1	
TAF13	MCM4	WT1	
UQCRC1	CLOCK	E2F2	
IL12RB1	IGF1	NR1H3	
MCM6	OCIAD1	TLX3	
NOS2A	TPO	E2F3	
ATP6V1H	ESR2	NFKBIB	
SELP	CDH1	SPIB	
ST6GALNAC3	MSR1	RAB11A	
EEF1G	ENG	DARS2	
ASPH	INPP5E	CDIPT	
PSMD1	PDE5A	GTF2B	
CFD	LTBP1	KALRN	
VAV3	NFKBIE	DBH	
LRRTM3	GAD2	GNG13	
NPPB	GATA4	GAB2	
ATN1	GNG3	PDE6C	
FASN	DAXX	EIF4EBP2	
NLK	AK1	EPHA2	
LCK	FTO	SLC5A3	
ABCA8	CDK10	NDUFA11	
ARFGEF2	SEL1L	HDAC11	
PIWIL1	ARRB1	AP3M1	
WAS	MEF2D	AZI2	
NOX4	CRTAM	ISLR	
CDC25C	LPIN3	ATP2B3	
ALDH1A1	IL1RAP	UGT1A10	
SEC22B	DDEF2	HOXD1	
CR2	SELE	C1QB	
TNFRSF10A	DDX23	SCN7A	

SKIP	IGF1R	AR
UTF1	LHX8	PMAIP1
PTGER1	HAGH	PAX2
MEFV	POLR2D	SETD2
PTGIR	FUT7	ETV5
HSPD1	CAPN10	F13B
ACTA2	SPTBN4	SREBF2
ALDH3A1	S100A12	HIBCH
CKMT1A	UPB1	CD93
ISG15	MKNK2	CDC27
SYN3	HS2ST1	GALNT12
ATP6V0C	DGKD	TBX21
GLI1	CCS	MCHR1
GK2	ATP1A2	BAD
CCR8	F2RL2	PDE11A
C14ORF166	CD2AP	GABRG1
FANCD2	IRAK1BP1	TRO
CLCA2	EEF2K	ADAM12
ETS1	CTSA	AGT
CCNC	BBS4	PLAT
ABCC4	LOC441996	PTGES2
HDAC2	GATA6	FLAD1
ABCA10	ADAR	GATA2
ITSN1	BCL2L1	LRIG1
DCI	H2AFY	TNC
JUNB	IARS2	VLDLR
IL1A	SFRP2	MLST8
GDF2	BMP15	PLA2G4C
THPO	RFNG	TH
AK5	NFS1	OAS1
CHN1	PTGER2	ACSL3
ATXN1	MUT	NMNAT2
ALKBH3	UXS1	CASP6
CCR6	GLG1	LMX1B
MAFF	ABCC2	GPI
ABCB7	FOXH1	NFKBIL1
PPAP2A	NR1H2	MX1
YWHAH	CHST15	SMAD7
NPY1R	HSP90B1	FGF10
DNAJC4	RETN	SARM1
ADH1C	LTBP4	SLC9A3R1

EIF4EBP1	SHC2	ZMYM2
CLEC7A	PINX1	SMN1
NGB	FHL2	CTNNB1
IFNA4	REM2	ATP5I
SCN2A	FTCD	GNAL
TUBD1	CAPN3	CHDH
KCNJ9	TFDP1	SDHA
JAK1	CLK1	GART
B3GALT1	GCG	GNB1
MAML3	LDHAL6A	C1GALT1
NCK2	APOBEC1	TWIST1
RPL6	CYP1A1	CD72
RUNX2	COMP	OXCT1
IFNK	SCN1B	RCHY1
ANXA5	WASL	UVRAG
SND1	CYP4B1	CA9
COL16A1	SLC22A18	MIOX
NT5M	BRUNOL4	TCF1
ARHGAP4	TDGF1	HTR1A
CNR2	ABCC6	NOD2
IL11	CHPF	ENTPD6
TAP1	NKD1	CDKN2D
ETV7	PSMF1	PIGL
ALCAM	OXCT2	NR1D1
PSMC1	FUT6	FMO4
PIAS4	TFRC	MECP2
GOSR2	MTFMT	DNMT3A
TFAP2C	NSD1	ATP6V1E2
TNFSF13	MALT1	RETNLB
CYSLTR2	ITPA	PRKACB
FUCA1	ITGAV	FGFR3
IFNA8	PNLIP	ALDOB
DGKA	GOT2	INSIG2
RDH5	ATP1B3	LMAN1
NUP214	METTL6	LTA
CLCF1	FANCL	SLC34A1
HIP2	HMGN1	CDC42
SFRP1	CTNNAL1	CAPRIN2
ARHGEF11	ERN2	C9
PALB2	EIF2S3	PELI1
HNF4G	DPYSL2	COL6A1

BTLA	GGT1	GHRL
MTNR1A	NMUR2	CTNNBIP1
AP2B1	GRHPR	SNAI1
ADORA2A	COQ3	CXCL2
CTSL2	HOXB4	ELSPBP1
IKBKAP	ATP1B2	BHMT2
HRG	YAP1	ATF7IP
RPL14	STMN1	PPARBP
DNAJA1	CXCR4	ANKFY1
FRAP1	IL17A	SOD2
FZD9	ACACB	LPIN1
TAPBP	SCP2	FHIT
NP	DES	BIRC3
MAPK13	ANKRD1	MPI
GPAM	NMUR2	IKBKE
DTNBP1	ERN2	SLC6A11
ALPP	IDI1	CLDN17
NOS1	ADRBK1	ABP1
CLDN22	GALNT10	CCL15
MLYCD	APEX2	GSS
POLR3H	FZD7	PDZK1
JUN	PLCB1	HES1
CBLC	CCNB3	CARS2
SLC2A3	GALC	FURIN
BCR	XCL1	NCOA3
COL13A1	IGFBP2	SPP1
A4GNT	A4GNT	DPF2
CD244	OPRD1	C4BPA
CD97	TCF7L1	AKR1B1
MAGEH1	YAP1	RDH14
PFKM	LRWD1	SMARCC1
HOXB6	BET1L	VNN2
NRG3	OLA1	PON1
ALDH2	ALDH2	ACTG1
ACER2	NRF1	CHST2
	HGFAC	SLC11A1
	APBA1	IRF4
	CHRNG	HCLS1
	HK2	KCNMB3
	NPR3	SNX4
	TBL1X	PKMYT1

SELL	UGT2B11
JPH3	CYP1A2
PIK3CD	
ZCCHC11	
ACCN1	
OTUB1	
WISP2	
PLAUR	
GNAI1	
CAPN9	
CDKL2	
CSF2RB	
HOXB4	
DIAPH1	
GGT1	
CAMK2G	
METTL6	
NRG3	
CRYAB	
NT5E	
CFLAR	
FZD10	
MADCAM1	
PDCD2	
YARS2	
CIRBP	
KLRC4	
ACER2	
POLR1E	
SERPINA10	
APOBEC3B	
GOT2	
AGXT	
EPOR	
NAIP	
RTF1	
BOK	
AKR1C3	
HOXB6	
IL8RB	
CD97	

CD28
PFKM
PTCRA
PSCD4
DBT
GABRE
GLP1R
APOC3
AOC3
AK3L1
ILK
ACPT
MFGE8
KDSR
PRDX5
CTNNAL1
COQ6
GRIA4
STAT5B
FZD8
CIDEB
GTF2H4
HADHA
MAGEH1
COQ3
ATP1B2
WDFY3
PTAFR
TGDS
CENTA1
TPH2
HMGN1
GNB2L1
OSBPL5
GSK3B
EIF2S3
COL13A1
GPR39
HPR
CYP2C19
ERCC5

SQLE
KLK7
PSME2
IL22RA1
RBM8A
ATP1B3

Supplementary Table S5. ERG-mediated migration shRNA screen GOrilla gene ontology.

GO Term	Description	p-value	FDR q-value	Enrichment
GO:0097720	calcineurin-mediated signaling	2.29E-05	4.65E-03	35.04
GO:0060592	mammary gland formation	8.10E-04	8.24E-02	35.04
GO:0031848	protection from non-homologous end joining at telomere	8.10E-04	8.30E-02	35.04
GO:0021910	smoothened signaling pathway involved in ventral spinal cord patterning	8.10E-04	8.37E-02	35.04
GO:0021775	smoothened signaling pathway involved in ventral spinal cord interneuron specification	8.10E-04	8.44E-02	35.04
GO:0021776	smoothened signaling pathway involved in spinal cord motor neuron cell fate specification	8.10E-04	8.51E-02	35.04
GO:0033173	calcineurin-NFAT signaling cascade	8.10E-04	8.58E-02	35.04
GO:0016340	calcium-dependent cell-matrix adhesion	8.10E-04	8.65E-02	35.04
GO:0043247	telomere maintenance in response to DNA damage	8.10E-04	8.72E-02	35.04
GO:0071108	protein K48-linked deubiquitination	2.19E-04	3.27E-02	21.02
GO:0006278	RNA-dependent DNA biosynthetic process	4.29E-04	5.34E-02	17.52
GO:0061036	positive regulation of cartilage development	1.81E-04	2.79E-02	12.74
GO:0048016	inositol phosphate-mediated signaling	2.66E-04	3.66E-02	11.68
GO:0032330	regulation of chondrocyte differentiation	2.66E-04	3.70E-02	11.68
GO:0061035	regulation of cartilage development	1.13E-04	1.84E-02	9.73
GO:0000723	telomere maintenance	1.50E-04	2.35E-02	9.22
GO:0032200	telomere organization	1.96E-04	2.99E-02	8.76
GO:0006302	double-strand break repair	3.44E-04	4.45E-02	6.18
**GO:0032479**	**regulation of type I interferon production**	**6.42E-04**	**7.15E-02**	**5.53**
GO:0006366	transcription by RNA polymerase II	5.01E-07	1.95E-04	5.49
GO:0051054	positive regulation of DNA metabolic process	3.46E-04	4.43E-02	3.69
**GO:0050727**	**regulation of inflammatory response**	**5.87E-06**	**1.42E-03**	**3.5**
GO:0051052	regulation of DNA metabolic process	2.70E-05	5.23E-03	3.44
GO:0000122	negative regulation of transcription by RNA polymerase II	1.10E-05	2.44E-03	3.2

**GO:0071396**	**cellular response to lipid**	**7.51E-05**	**1.34E-02**	**3.15**
GO:0045944	positive regulation of transcription by RNA polymerase II	1.26E-07	6.72E-05	3.08
**GO:0032496**	**response to lipopolysaccharide**	**2.86E-04**	**3.81E-02**	**3.08**
GO:0045934	negative regulation of nucleobase-containing compound metabolic process	4.27E-08	2.60E-05	2.93
GO:0019932	second-messenger-mediated signaling	4.78E-04	5.83E-02	2.92
**GO:0002237**	**response to molecule of bacterial origin**	**5.74E-04**	**6.62E-02**	**2.86**
GO:0051253	negative regulation of RNA metabolic process	1.10E-06	3.53E-04	2.84
GO:0045893	positive regulation of transcription, DNA-templated	1.15E-07	6.41E-05	2.69
GO:1903507	negative regulation of nucleic acid-templated transcription	1.44E-05	3.02E-03	2.66
